# Impact Response Features and Penetration Mechanism of UHMWPE Subjected to Handgun Bullet

**DOI:** 10.3390/polym16101427

**Published:** 2024-05-17

**Authors:** Yihui Zhu, Yang Song, Wei Wu, Jie Ma, Zhuangqing Fan, Yaoke Wen, Cheng Xu, Min Xia, Weifeng Da

**Affiliations:** 1 School of Automation, Nanjing University of Information Science and Technology, 219 Ningliu Road, Nanjing 210044, China; zhuyh90@nuist.edu.cn (Y.Z.);; 2 Jiangsu Key Laboratory of Big Data Analysis Technology (B-DAT), Nanjing 210044, China; 3 Inner Mongolia North Heavy Industries Group Limited Company Nanjing Institute, Nanjing 210049, China; 4 Daping Hospital, Army Medical Center, Chongqing 400042, China; 5 College of Science, National University of Defense Technology, Changsha 410073, China; 6 School of Mechanical Engineering, Nanjing University of Science and Technology, 200 Xiaolingwei Street, Nanjing 210094, China; 7 NingBo DaCheng Advanced Material Company Limited, Ningbo 315300, China

**Keywords:** UHMWPE laminate, ballistic damage, back-face features, SPH-FEM coupled model, energy transformation

## Abstract

Ensuring military and police personnel protection is vital for urban security. However, the impact response mechanism of the UHMWPE laminate used in ballistic helmets and vests remains unclear, making it hard to effectively protect the head, chest, and abdomen. This study utilized 3D-DIC technology to analyze UHMWPE laminate’s response to 9 mm lead-core pistol bullets traveling at 334.93 m/s. Damage mode and response characteristics were revealed, and an effective numerical calculation method was established that could reveal the energy conversion process. The bullet penetrated by 1.03 mm, causing noticeable fiber traction, resulting in cross-shaped failure due to fiber compression and aggregation. Bulge transitioned from circular to square, initially increasing rapidly, then slowing. Maximum in-plane shear strain occurred at ±45°, with values of 0.0904 and −0.0928. Model accuracy was confirmed by comparing strain distributions. The investigation focused on bullet-laminate interaction and energy conversion. Bullet’s kinetic energy is converted into laminate’s kinetic and internal energy, with the majority of erosion energy occurring in the first four equivalent sublaminates and the primary energy change in the system occurring at 75 μs in the fourth equivalent sublayer. The results show the damage mode and energy conversion of the laminate, providing theoretical support for understanding the impact response mechanism and improving the efficiency of protective energy absorption.

## 1. Introduction

In modern localized warfare, protecting the lives of military and police personnel is of paramount importance. Personal protective equipment such as bulletproof vests plays a crucial role in reducing soldier casualties. Researching how to provide more effective protection when encountering light weapon attacks is of great significance. Bulletproof vests with high ballistic ratings are already capable of effectively stopping small to medium-caliber rifle bullets while ensuring that back deformation does not cause severe injuries to the body. Therefore, studying the interaction between individual protection and projectiles, as well as the features of back deformation, is key to understanding the protective response to impacts.

High impact resistance, high abrasion resistance, high chemical resistance, flexibility, and excellent ballistic properties [1] make ultra-high molecular weight polyethylene (UHMWPE) fibers ideal for lightweight and high-performance body armor. Bulletproof undershirts are one of the applications of their flexible properties, and typically around 50 sheets of weftless fabric consisting of four layers of prepreg [2] stacked together can be used as soft body armor to meet the requirements of NIJ Level II ballistic standards [3]. Hundreds of layers of unidirectional prepregs are stacked together in a two-by-two orthogonal manner and hot-pressed to obtain UHMWPE laminates, which can be used as rigid inserts in composite protection. The laminate could be used as the flexible insert close to the human chest on composite bulletproof vests made of brittle ceramics and flexible laminates. The laminate can also be used as a helmet or to independently withstand the impact of handgun bullets.

For the study of the dynamic mechanical properties of UHMWPE orthotropic lay-up laminates, Shi [4] investigated the dynamic mechanical properties of UHMWPE orthotropic lay-up laminates with different lay-up angles under high strain rate compression and found that the energy absorption capacity varied with the lay-up angle and that the main damage modes were delamination and compaction in the thickness direction. Asija [5] utilized the SHPB test to evaluate the performance of the STF-treated UHMWPE composites at high strain rates and showed that STF-treated specimens outperformed untreated specimens in terms of peak stress, strain, strain rate, and impact toughness.

For numerical simulation studies on protective impact response, Long [6] proposed an improved Taylor approximation method. This new method utilizes the recursive relationship of the inverse Langevin function as an auxiliary tool, enabling a more accurate description of the relationship between stress, strain, and displacement in materials. Compared to the widely-used 5th-order Taylor approximation, this new method offers higher accuracy and stability when dealing with simulations involving large deformations. Nguyen [7] applied the equivalent laminate discrete method to accurately capture vertical failure. They simulated the impact of different thickness laminates, ranging from 12.7 mm to 102 mm, with calibers of 12.7 mm and 20 mm and velocities ranging from 400 m/s to 2000 m/s. They predicted penetration mechanisms and bulge morphology. Bürger [8] proposed a strain rate constitutive model to simulate the ballistic impact response of UHMWPE fiber-reinforced composites and predict energy loss. However, the overall modeling failed to simulate laminated damage and bulge height.

In terms of research on theoretical models for composite materials, Long [9] investigated the influence of temperature on critical energy release rates using damage mechanics material models and element deletion methods. By considering factors such as strain rate, temperature, and stress state, they simulated a three-dimensional fracture specimen using an advanced material model based on test data to find critical energy release rates at different temperatures. The simulation results showed that critical energy release rates increased with temperature. Numerically, the equivalence conditions in brittle and small-scale plastic fracture were proven [10]. Vaziri [11] proposed a model to predict the transient response of composite plates under non-penetrating impact. This model used two-dimensional elements to simulate the composite material but could not predict internal phenomena. However, it could predict the structural impact response. Analytical models for studying impacts only provide very limited solutions. This leads to the need for a large number of different analytical models for different scenarios. For example, Ben-dor [12] compiled around 280 analytical models, with 20 categorized as the most cited models.

The 3D digital image correlation (DIC) measurement and analysis method was first proposed by Luo [13]. Its basic principle combines the binocular stereo vision principle with digital image correlation matching technology to reconstruct the three-dimensional spatial coordinates of each point on the surface of the object before and after deformation. This enables the determination of surface morphology and three-dimensional deformation information. Compared to other optical measurement methods, 3D-DIC offers advantages such as non-contact, full-field measurement, automation, simplicity of optics, universality, and strong resistance to interference. As a result, it has been widely used in the mechanical property testing of various materials across different fields [14]. Currently, 3D-DIC technology is relatively mature. Freitas [15] and Bigger [16] have used high-speed photography with 3D-DIC to measure the penetration process of bullets of different calibers on various protective materials. They also calculated and analyzed the corresponding deformation. With the growing utilization of high-speed photography in the research of dynamic mechanical properties and impact response, 3D-DIC technology is gradually being applied in the mechanical property and impact testing of UHMWPE laminates.

In the research of impact response in fiber-reinforced composite laminates, the focus is primarily on investigating the influence of laminate thickness, impact velocity, and stacking sequence on penetration characteristics, local damage, and stress wave transmission. While 3D-DIC technology assists in capturing impact features, there is a need for further research on the response of thin laminates and analysis of strain distribution. Studying the strain distribution in UHMWPE laminates contributes to understanding the impact mechanisms and improving numerical models. Numerical simulations primarily aim to predict the dynamic mechanical properties of fiber-reinforced laminates, particularly the consistency of stress–strain curves with experimental data. However, global modeling fails to fully represent delamination phenomena, and the dynamic compressive behavior of interlaminar cohesive strength requires further investigation. Discrete modeling is suitable for laminates of varying sizes and thicknesses. Despite progress made by numerical models in studying back bulging, there is still a need to improve the simulation accuracy of strain distribution and compression wave propagation to deepen the understanding of deformation and damage mechanisms.

To address the limitations in current research, this study investigates the damage mode of UHMWPE laminate under typical pistol bullet impact and quantitatively analyzes the bulge morphology and strain distribution on the back surface using 3D-DIC technology. An optimized numerical simulation model is proposed, employing interlayer contact based on the same traction-separation criterion instead of cohesive units. This is because the large size of the laminate would require a significant amount of time when using cohesive elements. Although, since cohesive effects were not simulated with a mesh, it is difficult to determine the exact moment when interlaminar separation occurs. It has little influence on the focus of this study, which is more on the bulging and the morphological changes of the sub-laminates during the impact process. Therefore, this model is applicable for studying the impact response of laminates on a larger scale while appropriately reducing computational complexity without compromising accuracy. The validity of the numerical model is verified through comparison with experimental results, and further analysis is conducted to explore the interaction characteristics between the bullet and laminate, as well as the variations in energy. The experimental findings provide essential quantitative data for investigating the impact behavior of UHMWPE laminates, while the SPH-FEM-coupled numerical simulation method offers valuable insights into the interaction between pistol bullets and laminates.

## 2. Experiment Procedure

### 2.1. Materials and Apparatus

**Figure 1:**
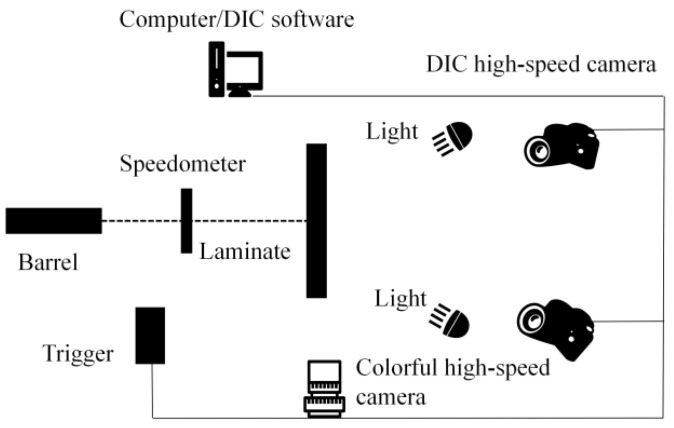
Schematic diagram of the testing system.

**Figure 2:**
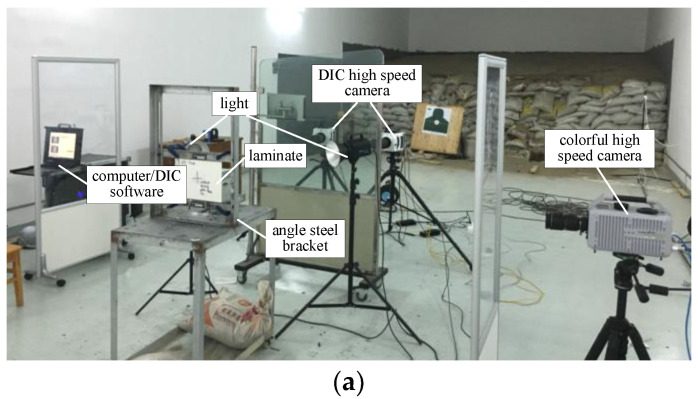

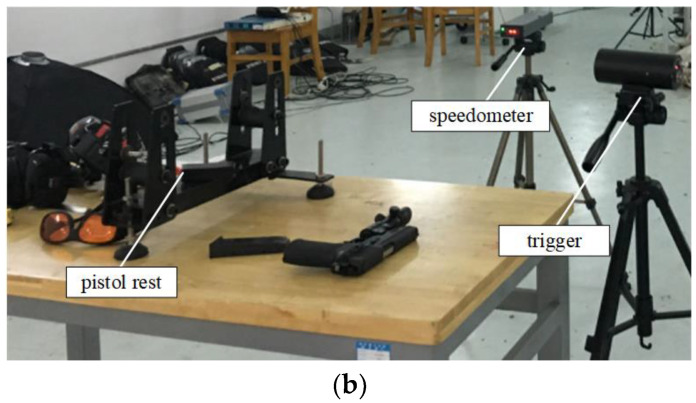
The site layout of the testing system. (**a**) Test System Panorama Diagram; (**b**) Firing Mechanism.

The test specimen was a UHMWPE laminate produced by Ningbo Dacheng New Materials Co., Ltd. (Ningbo City, China). The manufacturing process of the laminate involves the use of UHMWPE fibers with specifications of 1000D × 300F. The technical specifications of these fibers are a density of 0.97 g/m^3^, a tensile strength of 40 ± 2 g/D, a modulus of 1300 g/D, and an elongation rate of 3.3%. The production starts with the arrangement of unidirectional woven fabric in a bi-orthogonal pattern at 0°and 90°, followed by pressing in a vulcanizing machine at 80 ℃ for one hour to create a bulletproof fabric with a surface density of 160 g/m^2^. Subsequently, 55 layers of this fabric, each 0.2 mm thick, are stacked and hot-pressed for 1.5 h at 100 ℃ using a 1200-ton press to achieve the final shape. The resulting laminate measures 300 × 300 × 11 mm, has a surface density of 9.7 kg/m^2^, and the diameter of the fibers is approximately 25 μm. The matrix material used is water-based polyurethane, with a density of about 1.1 × 10³ kg/m^3^ and a volume fraction of around 20%. And the basic mechanical properties of the laminate are as follows: The average elastic modulus of the laminate is 25.7 MPa, the average tensile strength is 406.3 MPa, the tensile failure strain is 1.78%, the in-plane shear strength is 25 MPa, the maximum shear stress is approximately 158 MPa, the shear strain reaches 44%, and the punch shear strength is 158 MPa. The transient impact testing system for the laminates primarily consisted of DIC high-speed photography, color high-speed photography, a trigger device, and a velocimeter. The DIC high-speed photography was responsible for measuring the speckle displacement on the back of the UHMWPE laminate, which served as the target plate. This measurement provided information on the bulge height, width, and strain on the back. The color high-speed photography, positioned on the side of the target plate, compared the measured bulge height with the results obtained from DIC high-speed photography to validate the reliability of the DIC data. The velocimeter was used to measure the initial velocity of the projectile. The trigger device synchronized the DIC high-speed photography and the color high-speed photography. Schematic diagrams and on-site layout are shown in Figure 1 and Figure 2, respectively. The corners of the laminates were securely fastened using G-shaped clamps on angle steel brackets. The laminates were subjected to shots from NP22 9 mm lead-core bullets fired from a distance of 10 m, with an average initial velocity of 334.93 m/s.

### 2.2. Data Processing and Validation

**Figure 3:**
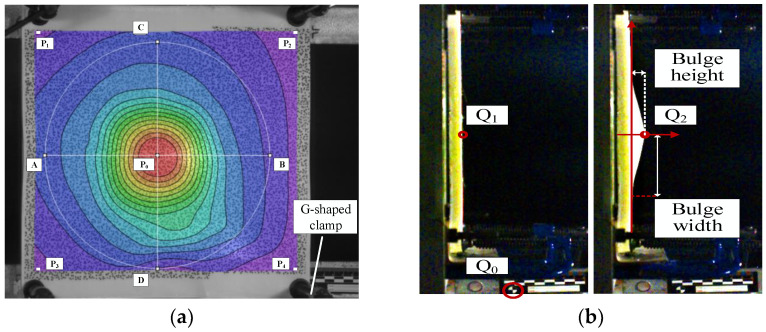
The positions of P_0_, L_AB_, L_CD_. (**a**) The positions of DIC; (**b**) The feature points on the side view.

Due to the variability of the clamping method and fixture, their influence on the bulge height of the laminated board needs to be considered. Figure 3a shows the clamping method, where G-shaped clamps are used to secure the four corners of the laminated board. The central point P_0_ is selected as the location of the maximum bulge height, from which displacement, velocity, and acceleration data for the maximum bulge height are extracted. Points P_1_ to P_4_ at the corners are used to extract the overall displacement of the laminated board in the z direction. The lines L_AB_ and L_CD_, which pass through point P_0_ in the x and y directions, respectively, are used to describe the variations of bulge width in the horizontal and vertical directions, thus depicting the changes in the bulge contour along the x and y directions.

Figure 3b illustrates the calibration of the side-view high-speed photography. A ruler is fixed to the fixture to calibrate the dimensions within the image. Point Q_0_ is selected to extract the overall displacement of the fixture in the z direction. Q_1_ represents the initial position of the maximum bulge height point, while Q_2_ represents its position at a certain moment. The difference between these two points is considered the displacement of the maximum bulge height point in the z direction. The bulge height and width are shown in Figure 3b.

**Figure 4:**
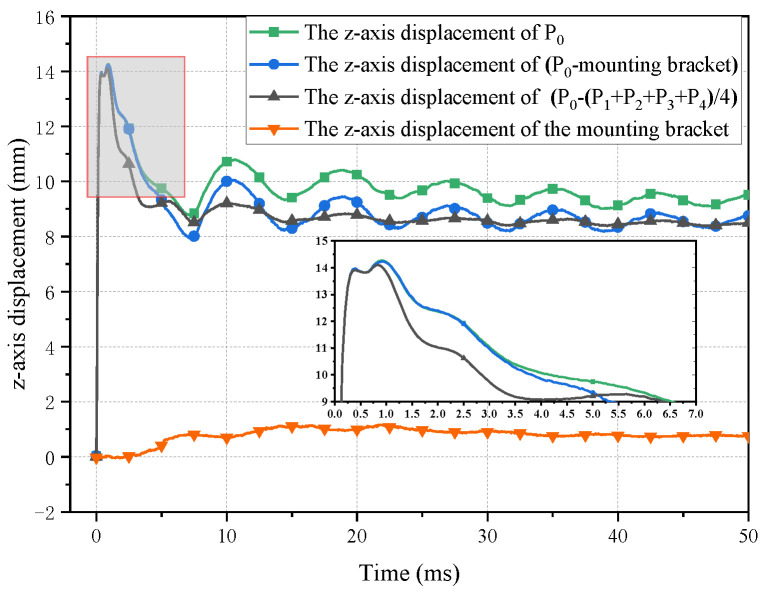
The influence of the bracket displacement and whole laminate displacement on the bulge height.

In addition to the intrinsic variation of the bulge height, the bulge height is also influenced by the movement of the fixture, as shown in Figure 4. The fixture is not fixed rigidly to the ground but is instead held in place by sandbags to prevent movement. It can be observed that after the fixture is subjected to impact, there is a gradual backward displacement. Furthermore, due to the limitations of the clamping device, the laminate may also experience displacement relative to the fixture. The fixture starts to move from 2.8 ms onward, without significant oscillation. On the other hand, the average displacement of points P_1_ to P_4_ indicates that the laminate undergoes overall displacement starting at 1.6 ms, with a relatively large amplitude of oscillation. The first peak occurs at 1.2 ms with a value of 14.23 mm. Therefore, both the displacement of the fixture and the overall displacement of the laminate have a minimal effect on the first peak. Regarding the second peak, the displacement of the fixture has some influence, though not significant. Specifically, when excluding the displacement of the fixture, the second peak occurs at 1.65 ms with a value of 14.26 mm. The difference between the two peaks is only 0.03 mm. Furthermore, the amplitude of the oscillation gradually decreases during the stable oscillation phase. In summary, the movement of the fixture and the overall displacement of the laminate have a minor impact on the values and trends of the two peaks. Therefore, in the subsequent data analysis, the data, without excluding the displacement of the fixture and the overall displacement of the laminate, are utilized.

**Figure 5:**
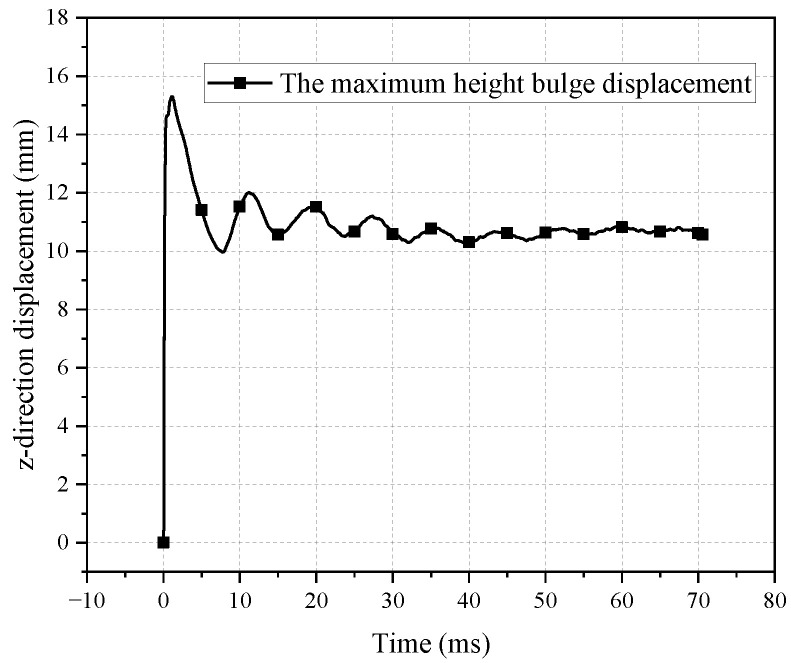
History of the maximum height of the bulge.

As shown in Figure 5, the maximum bulge heights all increase rapidly at first, followed by a second peak after a short fall, after which they fall rapidly and begin to oscillate.

Based on the data in Table 1, the second peak value of the bulge height obtained using the DIC method is 14.93 mm. In comparison, the peak value measured by side-view high-speed photography is 14.54 mm, resulting in an error of only 2.18%. After repeating the experiment twice, the test data showed similar errors to the data obtained in the first test. This indicates that the accuracy of the DIC analysis results has been adequately ensured.

## 3. Experimental Results and Discussions

### 3.1. The Morphology of Bullet-Impacted Surface

**Figure 6:**
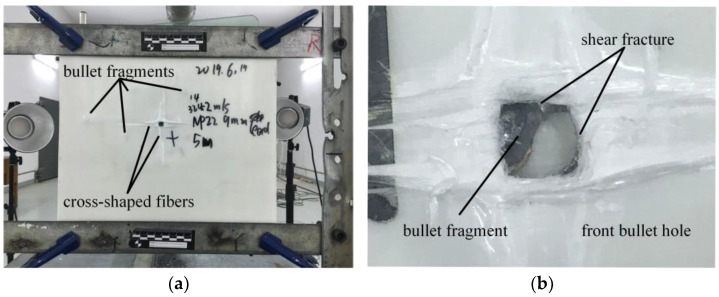

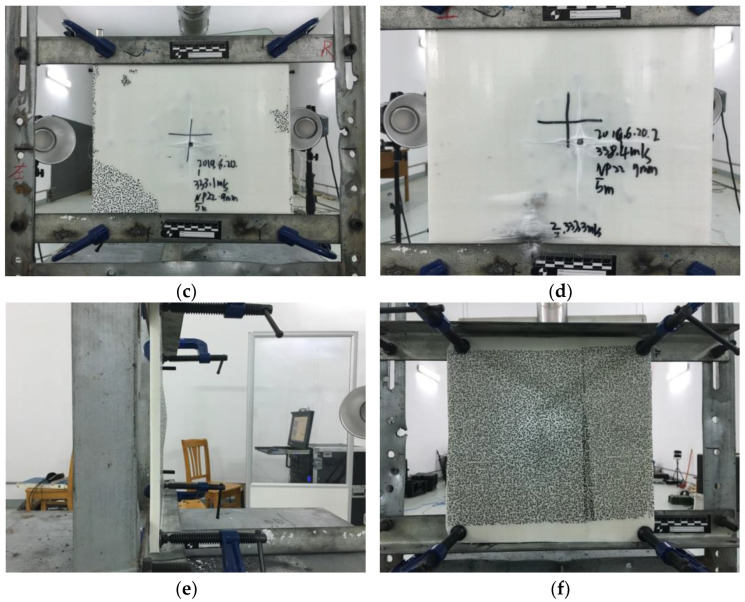
The impacted surface’s morphology. (**a**) The front-side morphology; (**b**) Bullet hole of Experiment; (**c**,**d**) The front-side morphology of repeat experiments; (**e**,**f**) The side-view and back side of the impacted laminate.

Figure 6 illustrates the morphology of the impacted surface. The laminated board can be clearly divided into two parts: the penetrated section and the unpenetrated section. The impacted laminate is initially subjected to compression and shearing by the bullet, specifically manifested as the fiber bundles in the x and y directions at the impact site being stretched under the effect of the bullet impact and the adhesive force of the laminate. Therefore, these fiber bundles are stretched before shear fracture and compressed to form a bullet hole. These fiber bundles then undergo elastic recovery after the bullet passes through the bullet hole. Moreover, since these fiber bundles have already partially detached from the main body of the laminate during the compression process, they exhibit a distinct cross-shape and detachment from the surrounding fibers after elastic recovery. There is a distinct layering between the penetrated layers and the unpenetrated layers. This could be attributed to the fact that the penetrated layers lose their effectiveness after being penetrated by the projectile. These layers relax and recover under the remaining tension of undamaged fibers, causing a separation from the unpenetrated layers that would have otherwise continued to displace under the influence of the projectile. This layering phenomenon between the penetrated and unpenetrated layers is similar to the transitional layer mentioned in reference [17]. It can be observed that there are only a few penetrated layers, and the layering in both sections occurs early in the failure process. Therefore, the penetrated and unpenetrated sections evolve independently, with the latter absorbing more energy [18].

It is evident that the bullet has shattered and dispersed into the inter-layer gaps of both the penetrated and unpenetrated sections, resulting in distinct cross-shaped patterns of fibers in the x and y directions. The extent of shell fragmentation is approximately 112.62 × 138.82 mm, while the cross-shaped pattern covers an area of approximately 130.00 × 132.35 mm. Cheeseman [19] mentioned that at the impact point, the predominant failure characteristic is the inter-layer shear between adjacent layers along the thickness direction, which is responsible for the formation of bullet holes. In contrast, the main failure feature observed here is the shear fracture between multiple fiber bundles due to the impact of the projectile, leading to the compression of surrounding fiber bundles that remain intact. Consequently, the originally regular arrangement of fiber bundles becomes distorted, resulting in the formation of cross-shaped fibers.

The morphology of the bullet holes is shown in Figure 6b. Each bullet hole exhibits a square-like shape with a very flat cross-section, indicating shear failure as the predominant failure mode. There is no significant layering between the penetrated layers, suggesting minimal relative movement between the layers, which reflects the good inter-layer integrity and bonding of the laminated board. As shown in Table 2, the dimensions of the bullet holes are 6.27 × 6.20 mm, smaller than the diameter of the projectile. This could be attributed to the coupling of shear and tensile stresses experienced by the fibers in the penetrated layers initially. After penetration, the tension in the fibers near the bullet hole is released, resulting in a reduction in the size of the bullet hole. The front-side morphology of repeat experiments is shown in Figure 6c,d. The side-view and back side of the impacted laminate are shown in Figure 6e,f.

### 3.2. The Morphology of the Back-Face Bulge

**Figure 7:**
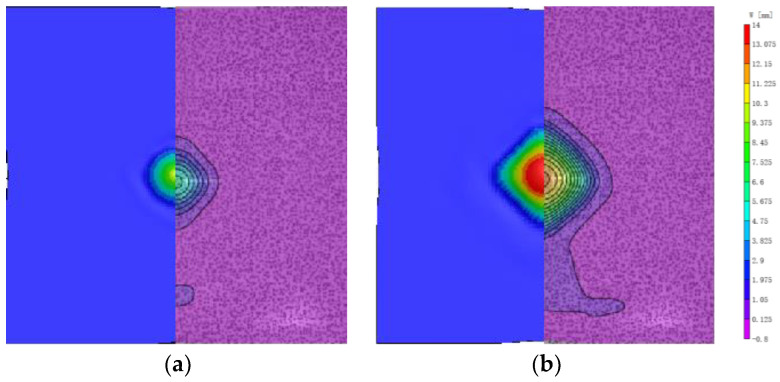

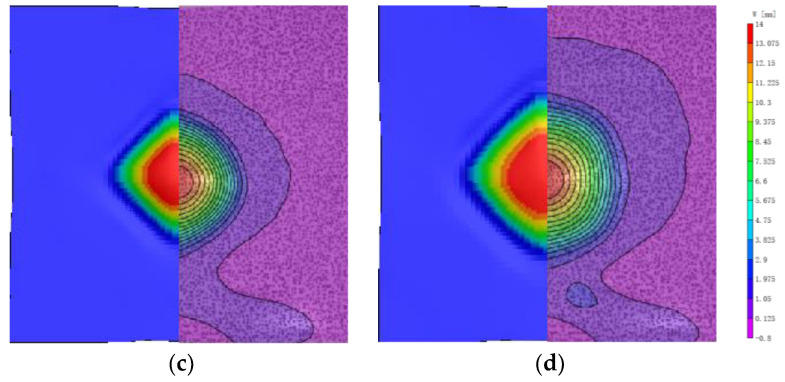
The bulge shape in z-direction (W). (**a**) 100 μs; (**b**) 200 μs; (**c**) 300 μs; (**d**) 400 μs.

Regarding the morphological changes of the bulge bottom surface, as shown in Figure 7. The simulation results are similar to the experimental results and are specifically analyzed in Section 4.2. When the laminate initially experiences impact, the fiber structure in the x and y directions receives the impact force first and absorbs energy. This leads to the fastest wave velocity in the x and y directions and results in a diamond-shaped appearance on the bulge bottom surface. As shown in Figure 7d, the propagation velocity along the 45° direction gradually exceeds that along the x and y directions, causing the impact response to continue increasing. The shape of the bulge bottom surface gradually transitions from a diamond to a circular shape. This may be attributed to the fact that the deformation of fibers along the x and y directions at the impact location is primarily caused by tension. Being in a high-modulus direction, deformation occurs rapidly. On the other hand, the deformation along the 45° direction requires transmission through friction between adjacent fiber bundles, which have a lower modulus, resulting in slower deformation. Additionally, at the four corners of the diamond shape, the impact force along the 45° direction causes the material to extend outward along the corners. Therefore, the diamond-shaped bulge bottom surface gradually transitions to a circular shape, indicating energy dispersion in the material.

After 300 μs, the rate of increase in the bulge height slows down, and the increment in the bulge width also decreases accordingly. As a result, the deformation in the x and y directions exhibits a reduced magnitude. However, as the bulge continues to grow, the accumulated energy needs to be released. The deformation in the x and y directions, constrained by their higher strength and modulus, becomes difficult to further increase. On the other hand, the matrix between adjacent fibers, with lower strength and modulus, can continue to deform to a certain extent. Consequently, the deformation along the 45° direction gradually increases, ultimately resulting in a square-shaped bulge on the bottom surface.

**Figure 8:**
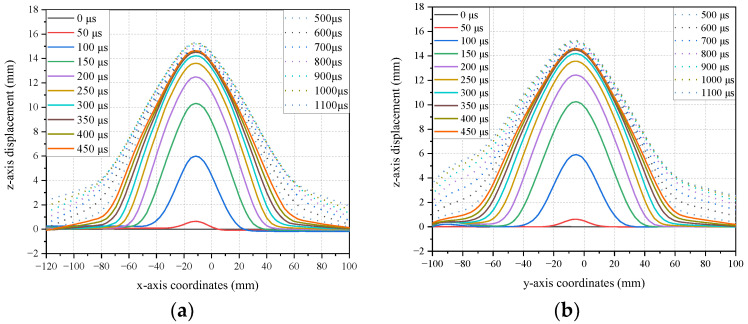
The profile of the bulge and its width. (**a**) The bulge profile in the x-direction along L_AB_; (**b**) The bulge profile in the y-direction along L_CD_.

The variation of the bulge profile along the x and y axes is shown in Figure 8. From 0 to 400 μs, the bulge height rapidly increases to reach the first peak. During this period, U and V also increase rapidly from 0 to 200 μs. As the bulge moves away from the impact center, the values of U and V initially increase and then stabilize at a fixed value, indicating that the fibers in the x and y directions rapidly converge towards the center of the bulge under tension. Correspondingly, as shown in Figure 7, the bulge exhibits a diamond-shaped morphology. After 200 μs, the rate of increase in the bulge height slows down, while the rate of increase in the bulge width accelerates, and U and V start to decrease. These characteristics indicate that the spreading speed of the bulge range exceeds the rate of height growth, and the fibers in the x and y directions begin to relax. Consequently, as shown in Figure 7, the bulge gradually transitions to a circular shape until it reaches 400 μs.

**Figure 9:**
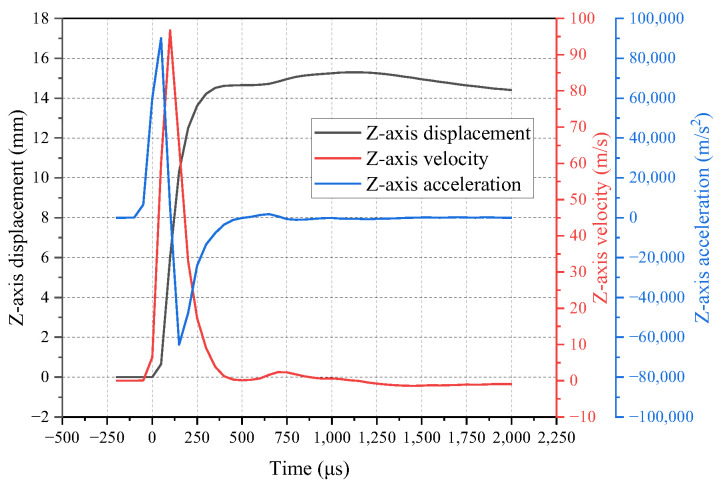
Displacement, velocity, and acceleration curves of the maximum point of the bulge height.

The variation of the highest point of the bulge height is shown in Figure 9. It initially increases rapidly and reaches the first peak at 14.61 mm at 400 μs. After a slight decrease, it starts to slowly increase again until reaching the second peak at 15.30 mm at 1150 μs, corresponding to the overall movement shown in Figure 8. Subsequently, the bulge height rapidly decreases. The maximum rate of increase in bulge height occurs at 100 μs, with a value of 96.68 m/s, while the maximum acceleration occurs at 50 μs, with a value of 901,650.20 m/s^2^, as shown in Figure 9.

**Figure 10:**
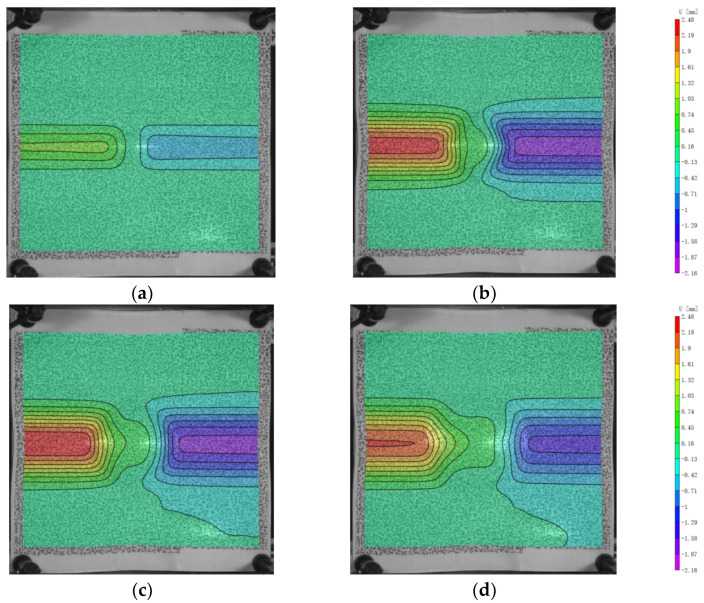
The displacement in x-direction (U) of the back face. (**a**) 100 μs; (**b**) 200 μs; (**c**) 300 μs; (**d**) 400 μs.

**Figure 11:**
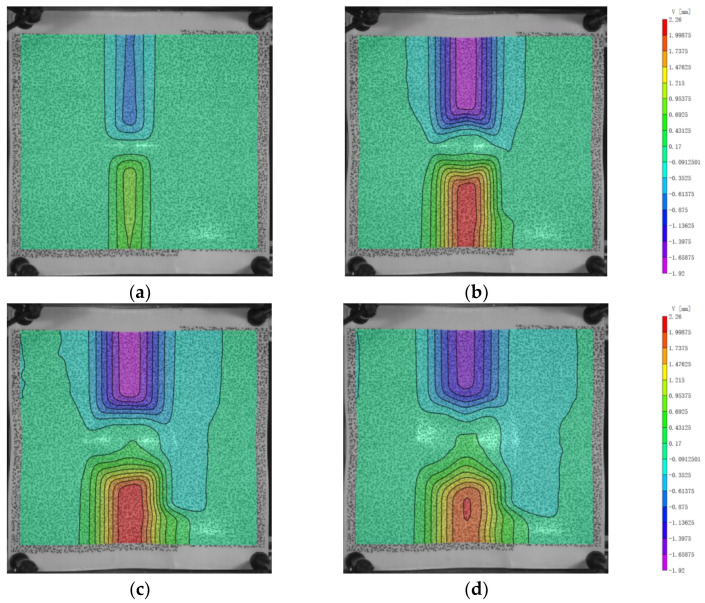
The displacement in y-direction (V) of the back face. (**a**) 100 μs; (**b**) 200 μs; (**c**) 300 μs; (**d**) 400 μs.

As shown in Figure 10 and Figure 11, the morphology of U and V initially indicates that the range of participation in deformation is similar for positions near and far from the impact center. Numerically, as the distance from the x and y axes increases, the values of U and V decrease. At 200 μs, the values of U and V increase as the positions get closer to the x and y axes. After 250 μs, as U and V decrease, the fibers farther from the center position recover faster, resulting in a larger recovery range compared to those closer to the center position in the experiment. This leads to a convex shape of the deformation range. Eventually, at 400 μs, the range of final recovery closer to the center position is smaller than that farther away from the center position. Thus, the range of participation in deformation is larger for positions closer to the center than for those farther away.

### 3.3. The Distribution of Strain

**Figure 12:**
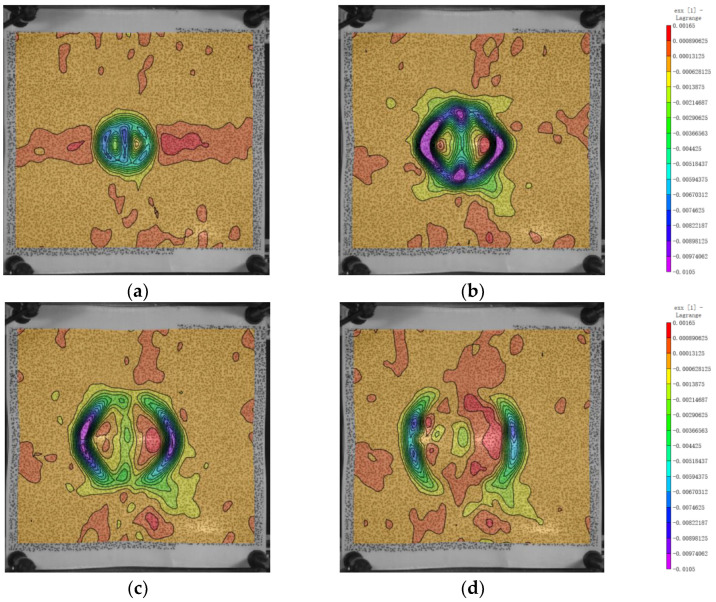
Evolution of the variable exx. (**a**) 100 μs; (**b**) 200 μs; (**c**) 300 μs; (**d**) 400 μs.

**Figure 13:**
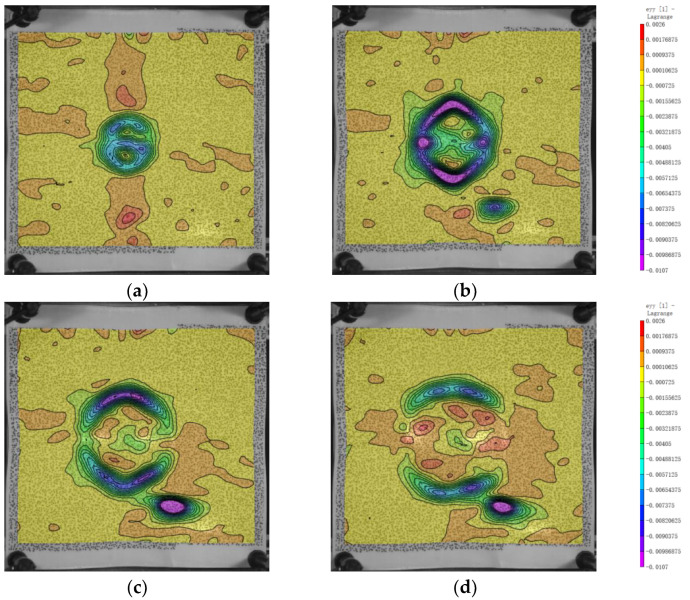
Evolution of the variable eyy. (**a**) 100 μs; (**b**) 200 μs; (**c**) 300 μs; (**d**) 400 μs.

The strain distribution on the back surface is shown in Figure 12 and Figure 13. Initially, along the x and y directions, the fibers at the bulge boundary experience an inward compressive force due to the impact, resulting in compression strain at the boundary fibers with a minimum value of −0.01 along the x and y axes. Over time, the stress propagates from the bulge boundary towards deeper regions, leading to tension in the middle fibers of the bulge. This causes an increase in the exx and eyy values, with a maximum value of approximately 0.0021. The magnitude of the minimum value is one order of magnitude larger than that of the maximum value, indicating stronger compression than tension. This reflects the instantaneous impact response of the projectile. As shown in Figure 12c,d and Figure 13c,d, from 300 μs to 400 μs, the strains in the x and y directions start to propagate outward from the impact location. With the dissipation of energy, the strain values near the impact location gradually decrease.

**Figure 14:**
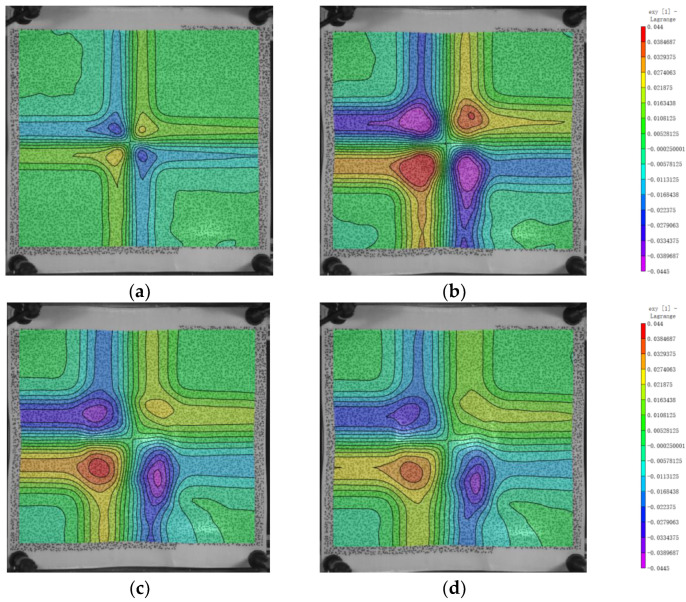
Evolution of the variable exy. (**a**) 100 μs; (**b**) 200 μs; (**c**) 300 μs; (**d**) 400 μs.

For the shear strain as shown in Figure 14, the minimum and maximum values of exy are around 0.0904 and −0.0928, respectively, and they occur at the boundary of the bulge in the ±45° direction. The shear strain is significantly larger than the strains in the x and y directions, indicating that the von Mises strain is mainly composed of exy. This suggests that the deformation of the laminated plate is much greater in the xy transverse direction than in the x and y directions. It can be observed that the x and y directions primarily bear the load from the fibers, while the xy transverse direction is supported by the matrix. This could be due to the significantly lower modulus of the matrix compared to the fibers, resulting in larger deformations and thus larger shear strains than strains in the x and y directions.

**Figure 15:**
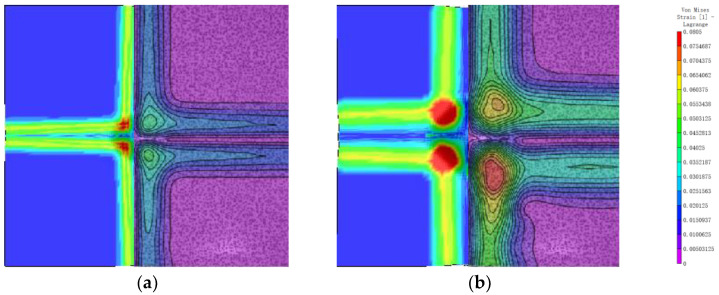

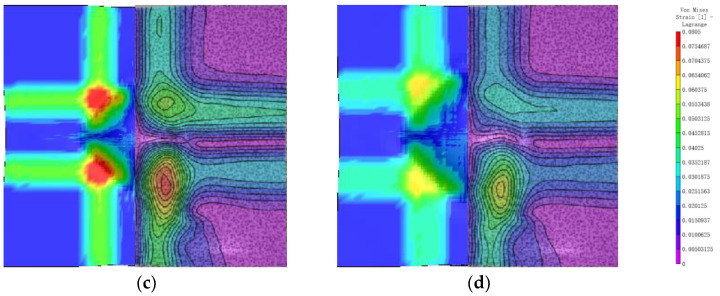
The evolution of the variable von Mises strain. (**a**) 100 μs; (**b**) 200 μs; (**c**) 300 μs; (**d**) 400 μs.

The von Mises strain field of the back surface is shown in Figure 15. The simulation results are similar to the experimental results and are specifically analyzed in Section 4.2. Taking the impact point as the origin and the x and y directions as the axes, the back of the laminate is divided into four regions, and the strain field distribution in each region shows an L-shape. This is due to the orthogonal layout of the laminate. The maximum value occurs at 200 μs and is 0.18. The maximum equivalent strain occurs at the boundary of the bulge, transitioning from the undeformed region to the deformed region.

**Figure 16:**
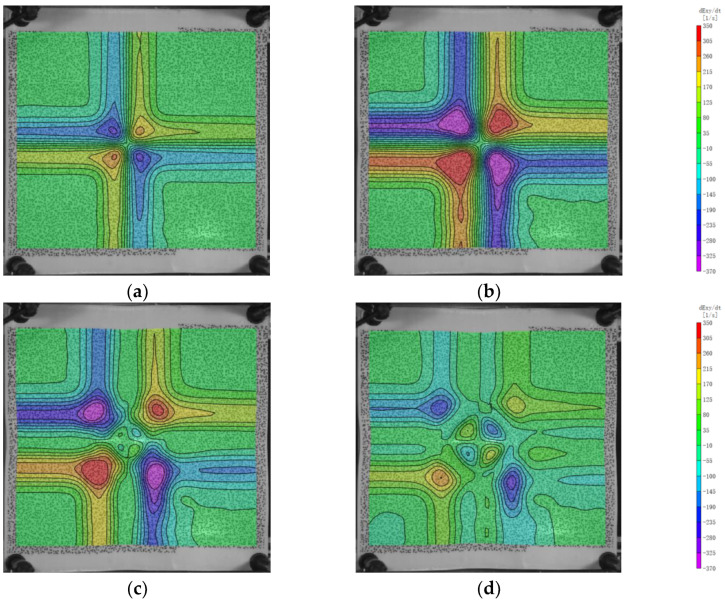
The evolution of the shear strain rate. (**a**) 100 μs; (**b**) 200 μs; (**c**) 300 μs; (**d**) 400 μs.

As shown in Figure 16, similar to exy, the distribution of the shear strain rate also exhibits an L-shape, with higher strain rates closer to the center. The maximum strain rate occurs at 100 μs, with a value of 808.65 s^−1^, and the minimum value is −840.09 s^−1^. Starting from 150 μs, there is a four-quadrant circular distribution of strain rates with reverse signs at the impact center, indicating a recovery state of shear strain in that region. With increasing time, the central recovery region expands, and the growth of shear strain at the boundary of the bulge gradually slows down, as shown in Figure 16d.

## 4. Numerical Model and Results

### 4.1. Finite Element Models

**Figure 17:**
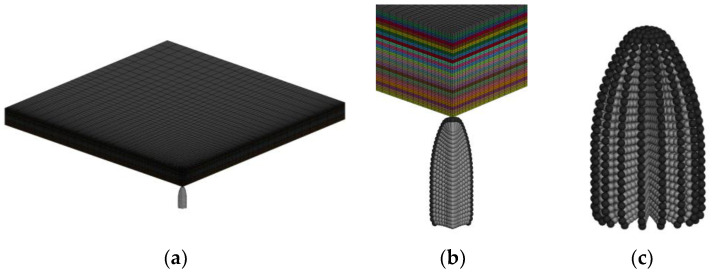
Numerical models. (**a**) Full model of the laminate; (**b**) 25 equivalent sublaminates; (**c**) full model of the bullet.

To investigate the main features observed in the experiments, numerical simulation models were tested for the impact response before 400 μs. The simulation employed a full-model numerical simulation with symmetric constraints. Figure 17a shows that the laminate has a denser grid in the impact region, and the overall laminate is simulated using equivalent sublaminates. Along the thickness direction (z-direction), the laminate is divided into 25 equivalent sublaminates, with 2 layers of grid points in each sublaminate. In the x and y-axis directions, there are 51 points on each face, resulting in 2500 elements per face and 5000 elements per equivalent sublaminate. To maintain accuracy and reduce computational cost, the contact relationship between adjacent equivalent sublaminate was handled using an automatic node-to-face contact based on traction-separation criteria, with contact being disconnected when contact option 9 was selected. An interface spacing of 0.001 mm was also set between adjacent equivalent sublaminates to avoid penetration. The bullet was simulated using the Smoothed Particle Hydrodynamics (SPH) method [20], as it is suitable for large deformations and does not require grid removal, allowing for simulating the bullet dispersion. Figure 17b shows the full model of the bullet. Contact between the bullet and the laminate was implemented using erosion node-to-face contact, with the soft 1 option opened with a coefficient of 0.2. Additionally, for boundary conditions, displacement in the x, y, and z directions was restrained for a 4 × 4 element grid on each corner of every equivalent sublaminates.

For the material model of the sublaminate, the MAT COMPOSITE FAILURE SOLID MODEL was used. Due to the correlation between the bulge shape and the shear modulus, as well as the correlation between the number of penetrated layers and the shear strength, the shear modulus Gab and shear strength were adjusted based on the height, width, and number of penetrated layers of the bulge observed in the experiments. Please refer to Table 3 for the specific material parameter values. Due to the laminated structure of the laminate, it can be considered orthotropic [5]. Therefore, the material properties in the 0°and 90°directions can be assumed to be the same. The commonly used Johnson–Cook material model was used to simulate the metallic materials [21]. Copper and lead were used for the bullet jacket and bullet core materials, respectively, and the Johnson–Cook material model was applied. Please refer to Table 4 for the specific material parameters.

To simulate the debonding process of the adhesive layer [23], the CONTACT_AUTOMATIC_SURFACE_TO_SURFACE_TIEBREAK contact algorithm was used between the equivalent sublaminate. In this contact algorithm, Option 9 was selected as a failure criterion. In this criterion, EN and ET represent the initial slope before normal and tangential separation, respectively, with units of stress/length. T and S represent the ultimate stresses in the normal and tangential directions, respectively. ΔN and ΔS represent the complete separation distances in the normal and tangential directions. Additionally, GIC and GIIC represent the energy release rates in the tangential and normal directions, respectively, with units of stress∙length. To ensure that the separation distance is greater than the unloading distance [17], the values of EN and ET should be sufficiently large. Please refer to Table 5 for the specific numerical values [24,25,26].

### 4.2. Numerical Validation

**Figure 18:**
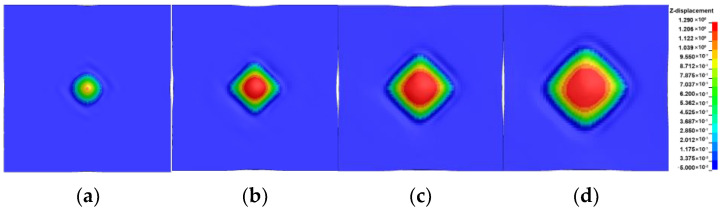
The evolution of the bulge morphology on the back surface: (**a**) 120 μs; (**b**) 220 μs; (**c**) 320 μs; (**d**) 420 μs.

The temporal evolution of the bulge bottom shape on the back surface, as obtained from numerical simulations, is shown in Figure 18. By comparing Figure 7 and Figure 18, it can be observed that the numerical model results are consistent with the experimental findings. Prior to 220 μs, the bottom shape of the bulge was diamond-shaped in both the simulations and experiments. In the experiments, the bulge bottom gradually transitions into a square shape, starting at 320 μs. The observed trend matches the experimental results.

**Figure 19:**
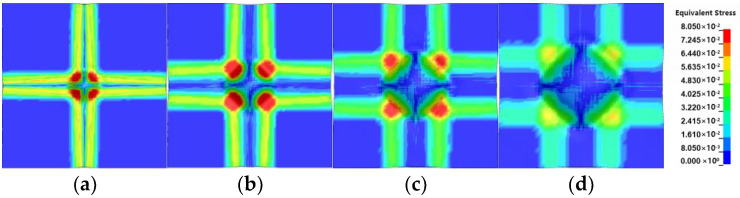
The evolution of the equivalent stress on the back surface: (**a**) 120 μs; (**b**) 220 μs; (**c**) 320 μs; (**d**) 420 μs.

Figure 19 depicts the evolution of the equivalent stress on the back surface. The overall trend is consistent with Figure 16. The stress wave is transmitted to the back surface at 40 μs after the initial impact. It can be observed that initially, the maximum equivalent stress is concentrated in the center and has a circular shape. At this stage, only a small region in the center experiences stress. Then, the equivalent stress along the x and y directions appears, gradually expanding towards the boundaries. During this process, the range of the maximum equivalent stress in the center also increases, and its value becomes larger. After 60 μs, the range of the equivalent stress in the center continues to expand, but the numerical values decrease. Additionally, the range of equivalent stress along the x and y directions gradually decreases. During this stage, both the bulge and the fiber direction experience stress. By 120 μs, the equivalent stress along the x and y directions disappears, while the range of equivalent stress in the center continues to expand and maintains a circular shape, with decreasing values. During this stage, the fiber direction no longer experiences stress, and the stress is mainly concentrated in the bulge. Subsequently, the center region of the equivalent stress gradually forms a diamond shape. By 420 μs, the equivalent stress magnitude on the four edges of the diamond shape surpasses that in the center. During this stage, the boundary region of the bulge experiences stress.

**Figure 20:**
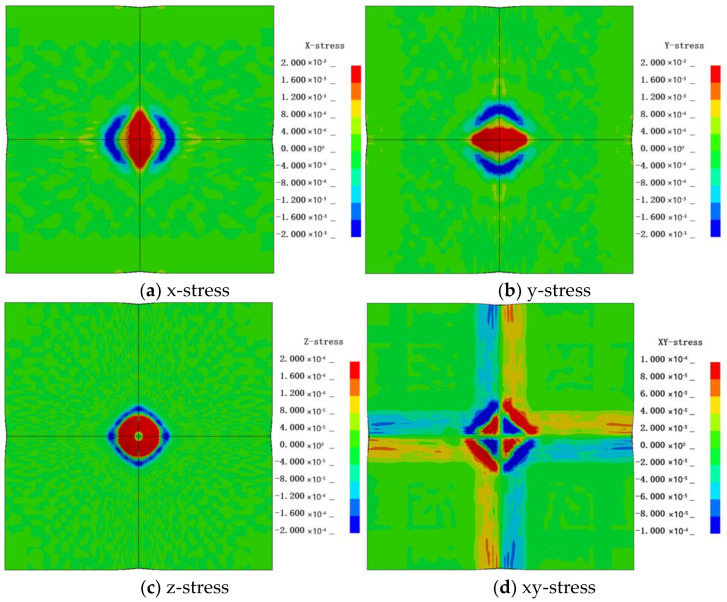
The stress of the back face.

Due to the inability to measure stress in experiments, only the stress distribution in the numerical model is analyzed. The analysis of stress results in the numerical model is showed as Figure 20.

**Figure 21:**
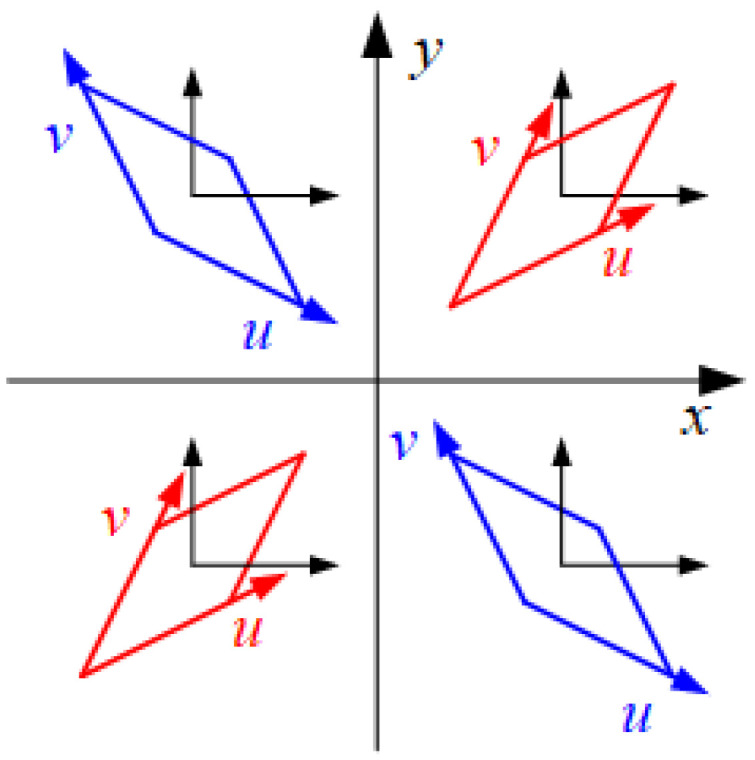
The shear strain exy represented by the deformation of the tiny unit.

The maximum and minimum values of x-stress are 1.828 × 10^3^ MPa and −1.507 × 10^3^ MPa, respectively, while for y-stress, they are 1.729 × 10^3^ MPa and −1.216 × 10^3^ MPa, respectively. It can be observed that the drumhead experiences tension in the middle, while regions near the x and y axes undergo compression at the boundaries. The stress distribution resembles the strain distribution depicted in Figure 12 and Figure 13. The maximum and minimum values of z-stress are 9.87 × 10^2^ MPa and −9.08 × 10^2^ MPa, respectively. At the center of the impact site, the stress is zero, with the drumhead sides experiencing tension and the boundaries experiencing compression. This indicates a certain expansion in the z-direction on the sides of the drumhead and compression at the boundaries in the z-direction. The maximum and minimum values of xy-stress are 6.50×10^2^ MPa and −6.72 × 10^2^ MPa, respectively. There is no xy-stress at the center of impact and along the x and y axes. The drumhead sides exhibit negative values, suggesting stretching along the 45° direction, while the boundaries show positive values, indicating stretching along the −45° direction. The directions of stress in the second and fourth quadrants are opposite to those in the first and third quadrants. The positive and negative values of strain are depicted in Figure 21.

**Figure 22:**
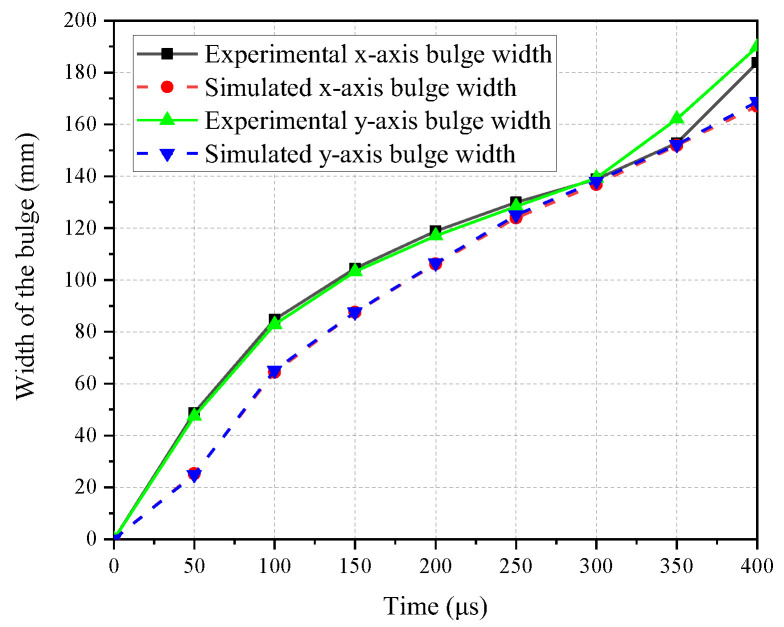
The evolution of the bulge width in the x and y directions.

The temporal evolution of the bulge width in the x and y directions, as observed in the numerical model and experiments, is shown in Figure 22. From 0 μs to 150 μs, both the experimental and numerical model results demonstrate a rapid increase in width. During the period from 150 μs to 300 μs, the rate of width increase slows down. However, in the experiments, the width of the bulge increases significantly after 300 μs, gradually transitioning into overall displacement. Correspondingly, in the numerical model, the rate of width increase after 300 μs also becomes larger, although it may not be as pronounced, still exhibiting a trend towards overall displacement.

The specific values are shown in Table 6. The numerical model and experimental results show minimal differences in the bulge width. The maximum relative error in the x direction is 24.12% and in the y direction is 21.29%. The minimum errors in the x and y directions are 1.49% and 0.85%, respectively. Additionally, both the experimental and numerical model results indicate that the width in the x direction is very close to the width in the y direction, demonstrating the orthotropic consistency of the material in the x and y directions.

### 4.3. The Morphology of the Bullet Impact Process

**Figure 23:**
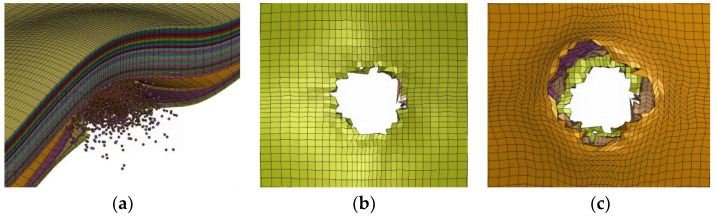
The morphology of the numerical model’s bullet hole at 400 μs. (**a**) 1/2 model sides; (**b**) front-side view; (**c**) back-side view.

The type of erosive damage to the bullet is stubbing. The type of erosive damage to the laminate includes penetration of the first three layers, partial damage of the fourth layer, and bulging expansion of the remaining parts. According to Figure 23, it can be seen that in the numerical simulation, the front four layers of the equivalent sublaminate are penetrated with a thickness of 1.35 mm, compared to the experimental result of 1.03 mm. The error is 25.18%, indicating that the numerical model is capable of replicating the observed bulge phenomenon in the experiment with reasonable accuracy. In the numerical model, the average diameter of the bullet hole along the x-axis is 7.78 mm, while along the y-axis it is 8.18 mm. Compared to the experimental data, there is an error of 19.23% in the x-axis direction and an error of 24.53% in the y-axis direction for the bullet hole diameter. Detailed data can be found in Table 7. A noticeable discrepancy between the numerical simulation and the experimental results is that the penetrated equivalent sublaminate does not attach together, as shown in the experiment, but exhibits separation. This separation may be related to the damage characteristics of the interlayer bonding force model used. In the experiment, the interlayer bonding force near the penetrated bullet hole is not completely lost, while in the initial penetration stage of the numerical simulation, the layers have already shown signs of separation, indicating an early failure of the interlayer bonding force. To reduce this interlayer separation, it may be considered to increase the distance parameter for complete separation between the layers, in order to better simulate the actual interlayer bonding behavior.

**Figure 24:**
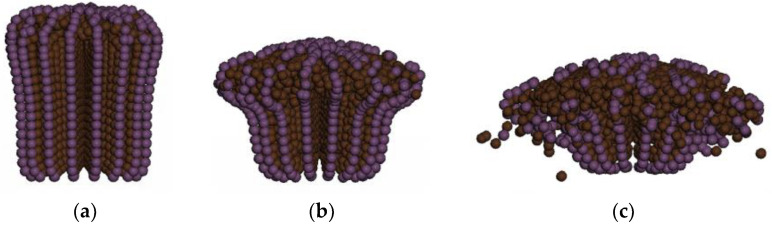
The deformation process of the bullet. (**a**) 50 μs; (**b**) 70 μs; (**c**) 90 μs.

Figure 24 illustrates the morphological changes of the bullet during the penetration process. Initially, the bullet head is stubbed at the beginning, and as the erosion process progresses, its front end gradually forms a mushroom shape, while the material at the rear end is compressed and concentrated forward. As the penetration continues, the front end of the bullet shell begins to fracture and disperse outward, while the material at the rear end diffuses around. Eventually, as shown in Figure 24c, the front-end fragments of the bullet core are scattered, but the main body remains concentrated near the impact point. The front end of the bullet shell fractures and disperses under impact, while the rear end forms several larger chunks.

**Figure 25:**
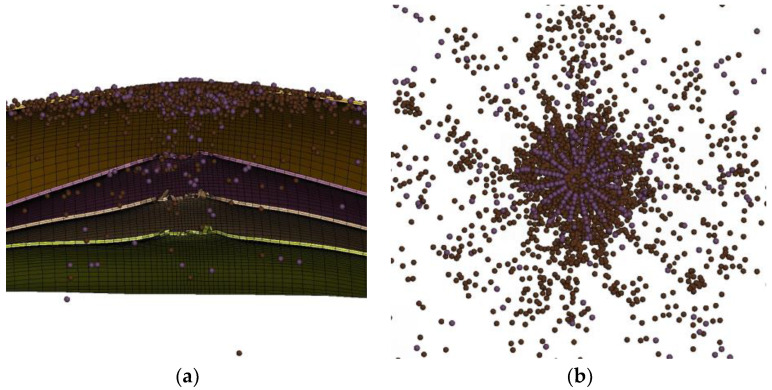
The top-down view morphology of the bullet at 400 μs: (**a**) side view; (**b**) upward view.

The simulated results are very similar to the observed deformation of the bullet core in the experiment, both exhibiting a mushroom shape. Due to the softness of the lead material, the main body of the bullet does not completely disperse. The bullet shell undergoes an unfolding process in both the simulation and the experiment. However, in the experiment, the bullet shell unfolds and splits into multiple small pieces, scattering and embedding into the laminate. In contrast, in the numerical model, the unfolded bullet shell only forms a few small pieces at the edges and does not completely fracture. This may be due to the bullet’s failure to fully penetrate the fifth layer, leading to partial penetration, and the fifth layer providing support to the bullet shell, restricting further unfolding and dispersal. Since the lateral splitting of the bullet was not restricted by the fifth layer, as shown in Figure 25, the bullet shell completely fractures into multiple small pieces and disperses in all directions after unfolding. Thus, it can be seen that further research on the interaction between the bullet and the laminates is needed to obtain more accurate simulation results.

**Figure 26:**
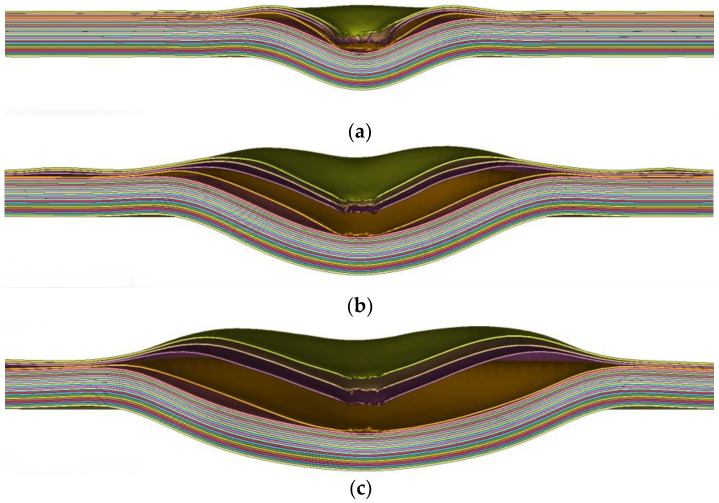

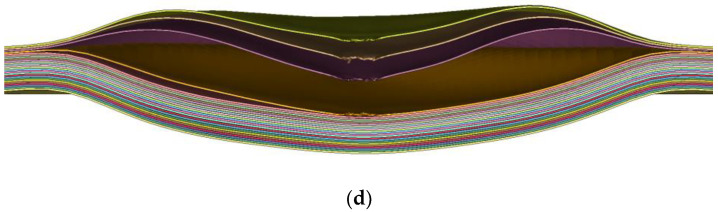
The evolution of the penetration response: (**a**) 100 μs; (**b**) 200 μs; (**c**) 300 μs; (**d**) 400 μs.

Nguyen’s [17] study pointed out that under the condition of impact velocity less than 500 m/s or laminate thickness less than 10 mm (in this study, the laminate thickness is 11 mm), the damage pattern does not include the shearing and stamping stage but only includes partial deformation penetration and the final bulging but not fully penetrated expansion stage, with partial deformation penetration being considered a transition stage. The specific details of the penetration process are shown in Figure 26, where the bullet starts to make contact with the laminate at 30 μs when it is a certain distance away from the laminate. During the time period from 40 μs to 100 μs, two significant phenomena were observed: first, a large bulge was formed on the laminate; second, there were noticeable traction marks on the edges of the laminate and along its x and y axes. As shown in Figure 26a, initially, the bullet compresses the laminate. Once compressed to a certain extent, under the action of compression along the thickness direction and around the edge, three sub-layers of the laminate are penetrated, as shown in Figure 26c. After penetrating three sub-layers, the bullet continues to move, causing local damage to the first sub-layer but not penetrating it. Subsequently, the penetrated three sub-layers have elastic recovery and separate from the unpenetrated parts. The unpenetrated parts continue to move with the impact of the bullet until the highest point is reached. The final shapes of the impact face and the back face are as shown in Figure 26d.

At 100 μs, partial fiber layers were observed to undergo penetration, indicating the occurrence of significant deformation penetration process. Subsequently, after 100 μs, the penetrated fiber layers began to partially recover and separate from the non-penetrated parts. Meanwhile, the non-penetrated parts continued to move under the propulsion of the bullet until approximately 400 μs, when the bulge reached its maximum height. This phenomenon corresponds to the characteristic of a protruding bulge that is formed but not penetrated. The experimental results also exhibit two main features: firstly, penetration occurred in approximately 1.35 mm of the fiber layers, with traction observed in both the x and y directions; secondly, the formed bulge ultimately remained unpenetrated.

### 4.4. Energy Transformation

**Figure 27:**
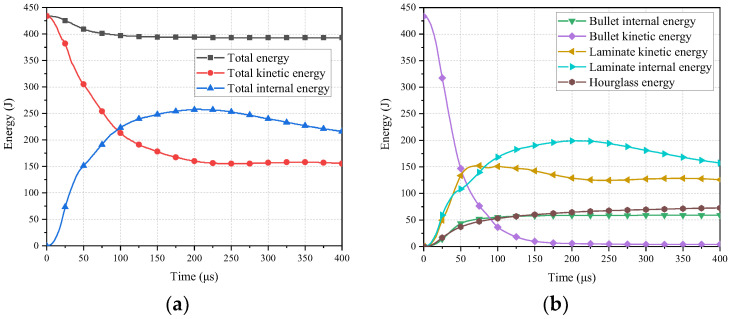
The evolution of energy: (**a**) The evolution of total energy; (**b**) The evolution of the laminate and bullet energy.

The energy distribution throughout the entire experimental process, as shown in Figure 27, reveals an initial kinetic energy of 433.4 J. As shown in Figure 27b, Before 150 μs, there is a rapid decline in the bullet’s kinetic energy, which is subsequently transferred to the laminate. At this point, the bullet’s kinetic energy is converted into both kinetic energy and internal energy within the laminate, leading to an increase in the laminate’s energy due to energy deposition. After 150 μs, the energy within the laminate marginally exceeds the changes in the bullet’s internal energy, likely attributed to sustained energy absorption and dispersion within the laminate’s larger volume. The variation in the bullet’s energy stabilizes around 200 μs, while the laminate’s energy remains at high levels, indicating a steady state in the late stage of energy transfer and absorption.

At the initial moment, the laminate begins to accumulate energy, possibly due to the formation and propagation of stress waves. By 71 μs, kinetic energy absorption within the laminate peaks at 152.18 J, subsequently trending towards stability, while internal energy absorption within the laminate continues to rise. This suggests potential structural changes or damage within the laminate. At this juncture, the bullet’s kinetic energy stands at 84.4 J, reflecting an 80.64% reduction, with the laminate absorbing 152.18 J of kinetic energy and 133.84 J of internal energy, while the deposited energy within the laminate amounts to 45.9 J. This further confirms substantial energy absorption by the laminate during the early stages of impact, in accordance with the principle of energy conservation.

At approximately 150 μs, the energy within the laminate begins to stabilize, and the energy curves of the system become parallel. This indicates a reduction in the rate of energy transfer, with the laminate likely dispersing energy throughout its entire structure. Subsequently, until 200 μs, the bullet’s kinetic energy remains nearly constant, indicating a stable state of energy transfer and absorption during the late stages of impact, while the energy within the laminate continues to sustain relatively high levels.

The first four equivalent sublaminates absorb a significant amount of energy and exhibit pronounced material erosion under the impact of the bullet. Additionally, compared to other layers, the mesh deformation in these first four equivalent sublaminate layers is more pronounced, possibly due to the propagation of stress waves and energy concentration during the bullet impact.

**Figure 28:**
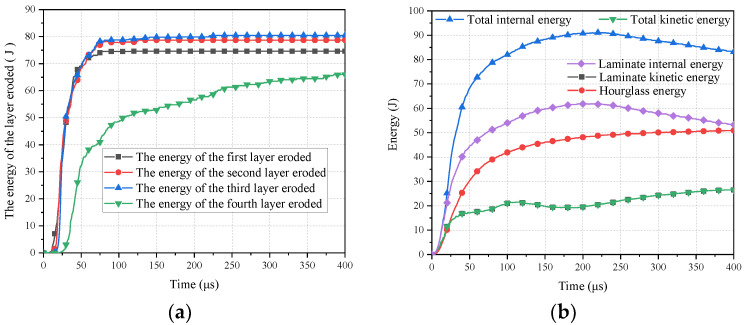
Energy of the equivalent sublaminate: (**a**) Eroded energy of the first 4 equivalent sublaminates; (**b**) Energy of the first 4 equivalent sublaminates.

As shown in Figure 28a, eroded internal energy is only present in the first four equivalent sublaminates, with no observed erosion in subsequent equivalent sublaminates. This indicates significant mesh deformation in the first four equivalent sublaminates due to erosion. This is because the first four sublaminates have been eroded and damaged enough to become bullet holes. The meshes in these areas become distorted and are deleted. Therefore, the erosion energy is present in these four sublaminates. The energy in the fourth equivalent sublaminate differs from the preceding layers, steadily increasing. At 75 μs, eroded energy stabilizes in the first three layers while indicating more significant variations in the fourth layer, consistent with the trend of the bullet’s kinetic energy dropping below the laminate’s internal energy observed in Figure 27.

According to Figure 28b, the internal energy of the first four equivalent sublaminates increases over time, peaking at approximately 200 μs before stabilizing. This suggests significant energy absorption in the initial stages by the first four equivalent sublaminates, consistent with the observation of the bullet’s energy depletion around 200 μs in Figure 27, indicating the completion of major energy conversion.

In terms of overall energy distribution, the hourglass energy constitutes only a small fraction, indicating that mesh distortion energy in the numerical model is not the primary source of energy dissipation, thereby confirming the accuracy and reliability of the numerical model.

## 5. Conclusions

This research applied the 3D-DIC technique to study the impact response of a 300 × 300 × 11 mm UHMWPE orthogonal laminate subjected to a 9 mm lead-core pistol bullet with a velocity of 334.93 m/s. An effective numerical calculation method was developed, which involved creating multiple equivalent sublaminate finite element models to simulate the impact characteristics of the laminate and reveal the energy conversion process. The experimental results demonstrated the bulging morphology and strain distribution on the back face of the laminate under impact. The numerical simulation results successfully corresponded to the experimental results and further investigated the process of bulging formation, the interaction between the bullet and the laminate, as well as the energy variation. The specific conclusions are as follows.

(1) Morphological characteristics of the back face: The penetration of a small number of fiber layers by the bullet is approximately 1.03 mm, accompanied by noticeable traction of the fibers near the x and y axes at the edges of the laminate, forming a cross-shaped failure feature. This is due to the compression and accumulation of fiber bundles.
(2) Bulge height and width: The bulge height initially increases rapidly, then the rate of increase slows down. The bulge width also initially increases rapidly, and then the rate of increase slows down. After 300 μs, the rate of increase in bulge height decreases, and the increase in bulge width also slows down accordingly.
(3) Strain distribution: In terms of trends, the location where the maximum shear strain (exy) occurs is approximately ±45° at the boundaries of the bulge. The location where the maximum tensile strains (exx and eyy) occur along the fiber direction is at the boundaries of the bulge along the x and y-axes, representing compressive strains. Numerically, the shear strain is larger than the compressive strain, indicating that the shear deformation of the fibers is greater than the compressive deformation. The maximum and minimum values of shear strain are approximately 0.0904 and −0.0928, respectively, while the maximum and minimum values of tensile strain along the x and y directions are approximately 0.0018 and −0.01, respectively.
(4) Validation of the numerical model: The corresponding numerical model can successfully simulate the numerical values and trends of the bulge height and width. By comparing the strain values at the same locations in the experiment and the numerical model, it can be seen that the numerical model results closely match the exx, eyy, and exy values at that location, both numerically and in terms of trends.
(5) Energy variation in the numerical simulation: The numerical simulation shows that the bullet impact rapidly reduces its kinetic energy, which is then converted into internal energy and the kinetic energy of the laminate. By 71 μs, the kinetic energy absorption of the laminate reaches a peak of 152.18 J, after which it stabilizes, while the internal energy absorption of the laminate continues to increase. The bullet’s kinetic energy decreases by 80.64% and is nearly converted into the kinetic and internal energy of the laminate. Significant erosion is observed in the first four layers of the equivalent sublaminates, and the energy is concentrated in these layers, indicating significant mesh deformation but overall reliability of the model.

## Figures and Tables

**Table 1:** The second peak value: 3D-DIC and side camera measured bulge height.

Experiment No.	Impact Velocity	The Maximum Bulge Height			Time
		3D-DIC	Side Camera	Relative Error	
	(m/s)	(mm)	(mm)	(%)	t (μs)
1	338.40	15.30	15.00	2.01	1150
2	333.10	14.26	13.89	2.70	900
3	334.20	15.22	14.73	1.83	1000
Average	335.23	14.93	14.54	2.18	—
SD	2.20	0.43	0.45	0.31	—

**Table 2:** The value of the morphology of the impacted surface.

Experiment No.	Penetrated Thickness	Bullet Hole		Shell Segment Diffusion		Cross-Shaped Fibers	
		x	y	x	y	x	y
	(mm)	(mm)	(mm)	(mm)	(mm)	(mm)	(mm)
1	0.95	6.15	6.11	113.21	145.21	136.42	127.62
2	1.06	6.34	6.33	110.32	124.46	133.22	139.35
3	1.07	6.32	6.14	113.67	139.41	131.57	141.11
Average	1.03	6.27	6.20	112.4	136.36	133.74	136.03
SD	0.05	0. 10	0. 12	1.82	10.71	2.47	7.33

**Table 3:** Laminate material parameters [21].

Parameters: Values	Parameters: Values
Density, ρ (kg/m^3^): 0.97	AOPT, MACF: 3.0, 1.0
Style modulus, E_a_, E_b_, E_c_ (GPa): 70, 70, 8	Tensile strength, X_T_, Y_T_, Z_T_ (GPa): 3, 3, 3
Poisson’s ratio, ν_ba_, ν_ca_, ν_cb_: 0.006, 0.06, 0.06	Compressive strength, X_C_, Y_C_, Z_C_ (GPa): 2, 2, 2
Shear modulus, G_ab_, G_bc_, G_ca_ (GPa): 0.05, 5, 5	Shear strength, S_ba_, S_ca_, S_cb_ (MPa): 700, 900, 900

**Table 4:** Warhead material parameters [22].

	ρ	G	A	B	N	C	M	T_m_	T_room_	C_p_	D_1_	D_2-5_
	kg/m^3^	GPa	MPa	MPa				K	K	J/(kg·K)		
Lead core	11.34	7	14	18	0.685	0.035	1.68	600.0	294.0	126	1.0	0
Headshell	8.45	46	90	292	0.01	0.025	1.09	1356.0	300.15	383	0.8	0

**Table 5:** Tiebreak face contact parameters [27,28].

Parameters: Values	Parameters: Values
Normal strength, T, (MPa): 1.0	Tangential strength, S (MPa): 1.6
Normal stiffness, E_N_ (MPa/mm): 400.0	Tangential stiffness, E_T_ (MPa/mm): 689.0
Normal energy release rate, G_IC_, (MPa·mm): 0.5	Tangential energy release rate, G_IIC_, (MPa·mm): 1.016
Normal maximum limit distance, Δ_N_: 1.0 mm	Tangential maximum limit distance, Δ_S_: 1.27 mm

**Table 6:** The bulge width in the x and y directions at various time in the experiments and numerical model.

Time	Exp. x	Num. x	Relative Error	Exp. y	Num. y	Relative Error
(μs)	(mm)	(mm)		(mm)	(mm)	
100	84.82	64.36	24.12%	82.81	65.18	21.29%
200	118.89	106.16	10.71%	117.00	106.50	8.97%
300	138.87	136.80	1.49%	139.28	138.10	0.85%
400	183.77	167.03	9.11%	189.97	168.74	11.18%

**Table 7:** The size of the bullet hole in the numerical model.

Penetrated Layers	The Size of the Bullet Hole in the Numerical Model	
	x (mm)	y (mm)
1	8.75	7.53
2	7.86	8.31
3	7.67	8.81
4	6.82	8.05
Average	7.78	8.18
SD	0.45	0.32
Relative error	19.23%	24.53%

## Data Availability

The data presented in this study are available on request from the corresponding author due to privacy and contractual limit.
